# Multisystemic Benign Metastasizing Leiomyoma: An Unusual Condition with an Atypical Clinical Presentation

**DOI:** 10.1155/2019/7014248

**Published:** 2019-04-10

**Authors:** Fernando Matos, Cristina Santiago, Duarte Silva

**Affiliations:** Radiology Unit, Centro Hospitalar Tondela-Viseu, EPE, Portugal

## Abstract

Benign metastasizing leiomyoma (BML) is a rarely found entity with few documented cases in the literature, usually occurring in women of reproductive age with a history of myomectomy or hysterectomy. The leiomyomas can metastasize to several organs, the lungs being the most commonly affected. We report a case of a 40-year-old female patient who presented at our institution with low back pain. She underwent a lumbar MR that revealed the presence of an expansive and compressive mass in the body of L4. This mass was biopsied, corresponding to a metastasizing leiomyoma with no malignant characteristics. Subsequently, a CT examination showed several soft tissue density round masses in both lungs, but the most striking feature was a 12 cm mass located in the left iliac crest. There was asymmetrical uterus enlargement, caused by the presence of several leiomyomas. Since the lesions were estrogen and progesterone positive, hormone suppression consisting of oophorectomy followed by anastrozole was the chosen treatment. No signs of progression were observed at the 6-month follow-up. This case is one of the very few that occurred in a woman with no previous uterine intervention, adding further evidence that surgery is not an essential condition for this entity to develop.

## 1. Introduction

BML is a rarely found condition that affects women with a history of uterine leiomyomas. Steiner first described BML in 1939 in a report of a patient who died from the effects of extensive pulmonary metastasis that were histologically identical to multiple leiomyomas concurrently present in the uterus [1].

BML usually occurs in women of reproductive age that present, most of the times, with a previous history of myomectomy or hysterectomy [2]. The leiomyomas can metastasize to several organs, the lungs being the most common target. In very few cases, other systems or organs, such as the skin, bladder, esophagus, liver, and bone, can also be affected by the condition [2, 3]. Although BML has typically benign histological characteristics, the fact that it can metastasize suggests a malignant component. Due to this ambiguity, there is still no consensus about etiology and treatment.

## 2. Case Presentation

**Figure 1:**
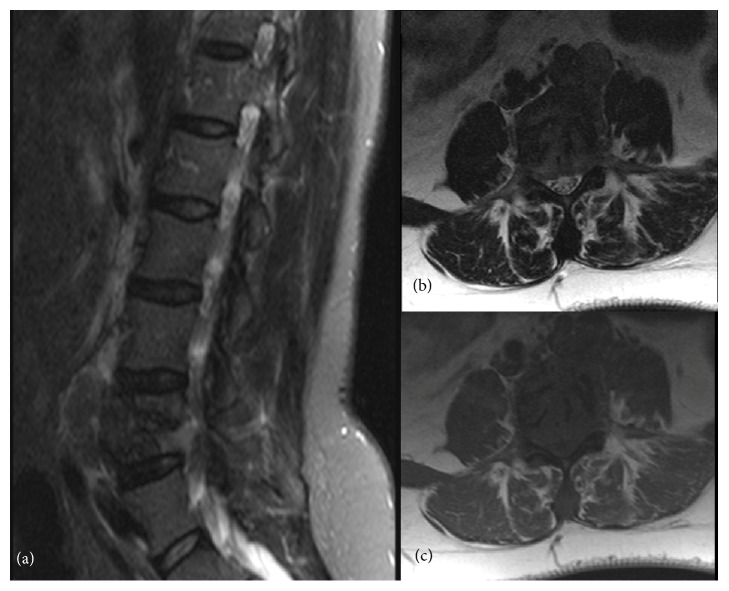
MR of the lumbar spine. (a) T2-weighted sagittal Spectral Attenuated Inversion Recovery (SPAIR) image. (b) T1-weighted axial image. (c) T2-weighted axial image. There is a T1-hypointense, T2-heterogeneous soft tissue mass occupying and destroying a large part of L4, causing nerve root compression.

A 40-year-old female patient presented at the Emergency Department of our institution with complaints of back pain for the last three days that started after moderate physical exertion. The patient referred no pain relief after taking anti-inflammatory drugs and denied respiratory symptoms such as chest pain, dyspnea, or cough. No weight loss, anorexia, or other symptoms were reported. There was no referral of previous surgeries or medications. A different, stronger anti-inflammatory drug was prescribed, but three days later she returned to the hospital, where a lumbar radiograph revealed the presence of a lytic lesion in the L4 vertebra. Due to the nonspecific appearance of the lesion, the patient underwent an MR of the lumbar spine. The exam revealed the presence of a heterogeneous, T1-hypointense mass in the body of L4, causing its partial destruction, and nerve root compression (Figure 1). A CT-guided biopsy was performed in order to assess the etiology of this mass. The pathology report described the presence of tumor fragments of mesenchymal origin with smooth muscle differentiation that were diffusely positive for estrogen and progesterone receptors. No obvious nuclear atypia or mitotic figures were identified. Ki-67 proliferation index was less than 1%. The final report stated that the lesion was compatible with BML.

**Figure 2:**
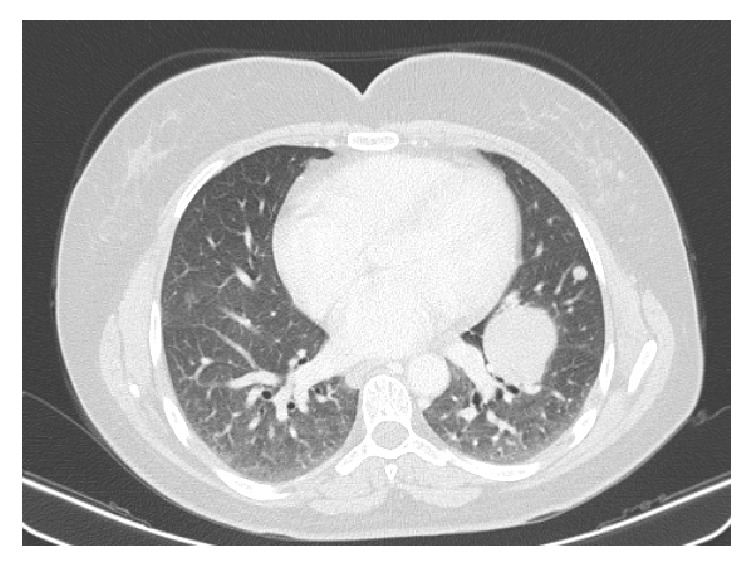
Chest CT, axial image. There is a soft tissue density mass measuring 44 mm and an 8 mm nodule, both in the left lung.

**Figure 3:**
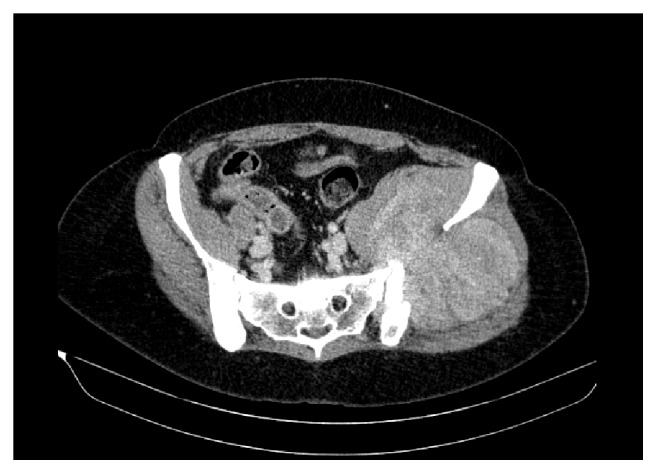
Contrast-enhanced CT. Axial view. There is a heterogeneous enhancing mass destroying the left iliac bone and expanding both in and out of the pelvic cavity. The iliac vessels have deviated towards the right. No evident infiltration of pelvic structures was observed.

**Figure 4:**
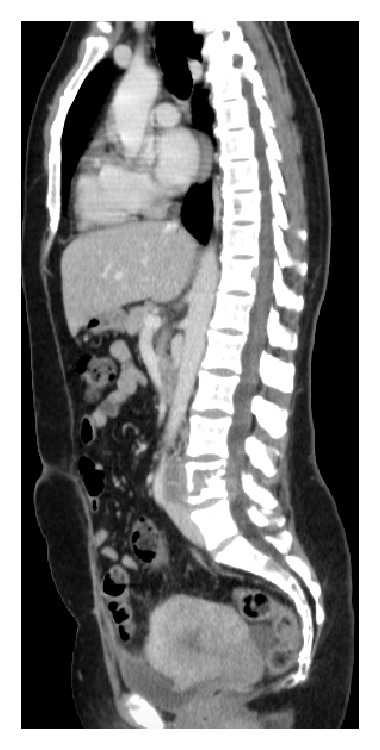
Contrast-enhanced CT. Sagittal view. There is a lytic lesion occupying the L4 vertebral body displaying a soft tissue component. The uterus is enlarged and depicts a nodular and lobulated contour, due to the presence of several leiomyomas.

Subsequently, a contrast-enhanced CT was performed to evaluate if other organs were affected: there were several soft tissue density round masses in the thorax, the largest being located in the left lung, measuring 44 mm (Figure 2). There was a 12 cm mass in the left iliac crest that enhanced after intravenous contrast. This mass had a lytic component and exhibited an intrapelvic bulky element (Figure 3). There was enlargement of the uterus due to the presence of several leiomyomas (Figure 4).

**Figure 5:**
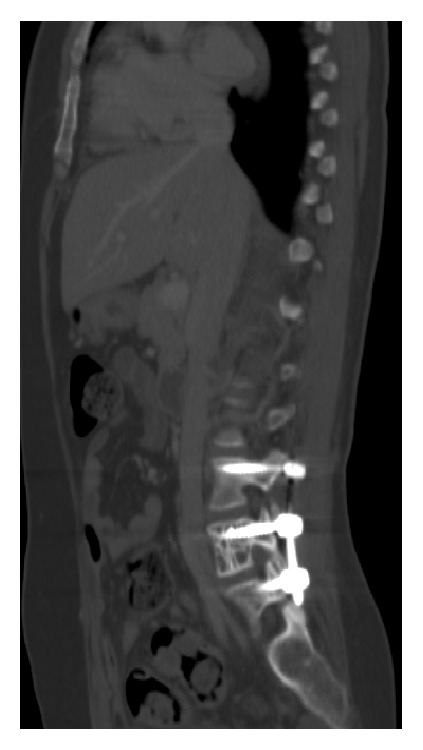
CT. Sagittal view. Pedicle screw fixation of L3-L5 after subtotal tumorectomy.

The clinical conduct included vertebral subtotal tumor removal, laminectomy, and pedicle screw fixation on L3-L5 (Figure 5) to decompress the nerve roots and reduce the symptoms. Oophorectomy and hysterectomy were performed and confirmed the benignity of the leiomyomas. Outpatient treatment consisting of anastrozole, an aromatase inhibitor, was prescribed. At the 6-month follow-up CT examination, the nodular pulmonary and iliac crest masses kept the previous dimensions and no new lesions were observed. Long-term follow-up was recommended in this case.

Regarding the low back pain, the patient referred a moderate improvement. Despite this, she needed to resort frequently to taking anti-inflammatory drugs after some physical exertion. The patient also reported a sensation of pressure in the pelvic region, especially in the lateral decubitus, most likely associated with the mass of the iliac bone.

## 3. Discussion

The causes of BML remain, in part, unexplained. On the one hand, BML is more frequent in women submitted to a previous myomectomy/hysterectomy, corresponding to the vast majority of the published cases to date [4]. Peritoneal seeding after myomectomy or hysterectomy for uterine leiomyomas is a possibility that would explain the increased incidence of BML in this group of women [4]. However, the reports on the seeding hypothesis are scarce [5], and there are currently no studies that support this possibility.

On the other hand, the reports of BML occurring in women with an intact uterus imply that a previous uterine intervention is not an essential condition for the development of this entity. Therefore, there must be a cause, other than the mechanical spread of cells, that would explain the origin of these metastasizing leiomyomas. One hypothesis is that BML is related to metaplasia of the coelomic epithelium [6]. According to this theory, the mesenchymal cells undergo alterations in the differentiation process, becoming differentiated erroneously into myofibroblasts. Another described entity, intravascular leiomyomatosis, consisting of smooth muscle cells growing through uterine venules has been shown, through genomic hybridisation studies, to share similar characteristics with BML according to the study performed by Lee et al. [7].

This case report is one of the very few that exhibits unusual clinical and radiological characteristics for BML: the fact that the patient was symptomatic at the time of diagnosis, findings in both lung and bone, and the absence of previous hysterectomy/myomectomy. By reviewing the current literature, there are, as far as we know, seven BML cases in women with an intact uterus. The most frequent (and unique) location of the metastasis of leiomyomas was the thorax [8–12]. One BML case [13] was multisystemic (bone, lung, and lymph nodes) in a woman with a renal transplant taking immunosuppressant drugs. One case [9] reported the simultaneous presence of lung lymphangioleiomyomatosis and BML and the possibility of a common etiopathological ground for both conditions. The forms of presentation varied from asymptomatic [10, 12, 14], respiratory [9, 11], or neurological symptoms [13]. Only one case [13] presented an exuberance of the imaging findings comparable to the one we describe.

Also noteworthy is the rarity of spinal BML, having only 10 cases been reported to date [14]. Diagnosis by imaging alone is almost impossible due to its rarity [14] and features that may overlap with other entities.

Differential diagnosis plays a crucial role in BML. Malignancies, such as low-grade leiomyosarcoma, should be excluded through the evidence of lack of atypia or necrotic areas [15]. It is not possible to histologically differentiate a BML from a primary uterine leiomyoma.

Treatment is not standardized due to the rarity of this entity. Several clinical approaches have already been adopted such as watchful waiting, surgical excision [16], oophorectomy [16], and the use of aromatase inhibitors [17]. Suppression of estrogen production was the main goal in this case since the lesions displayed estrogen and progesterone receptors. This was achieved by primarily performing the oophorectomy. The use of anastrozole permitted further reduction of peripheral estrogen production in order to maximize the possibilities of shrinkage of the masses. There were no changes in the dimensions at the 6-month follow-up examination. According to the existent literature, lung lesions tend to remain stable [18] with occasional regression after treatment [17].

## 4. Conclusion

In this case report, we reviewed a rare case of BML developing in the bone and the lung, in a patient that had not been submitted to a previous hysterectomy. While histological studies were essential to confirm the diagnosis, imaging played a key role in localizing and monitoring of the lesions. Both the referring doctor and the radiologist should be alert to this diagnosis when a woman with a history of leiomyomas and multiple pulmonary nodules presents at a given context. In this case, this was not as easily assessable due to unique confounding factors. The treatment, in this case, was based on hormone production suppression, with no signs of progression at the follow-up. Given that BML may have several different clinical presentations and progression patterns, an individual approach should always be taken into account.
